# Prevalence of Intestinal Amebiasis Among Saudi Residents Living in the Al-Baha Region, Saudi Arabia

**DOI:** 10.7759/cureus.94786

**Published:** 2025-10-17

**Authors:** Ashraf G Tımsah, Ibrahim Shatla, Mohammed M Alzahrani, Abdulaziz A Alzahrani, Faisal M Alzahrani, Osama A Alghamdi, Muhannad M Alghamdi, Saeed Alghamdi, Adel K Alghamdi, Majed A Alghamdi, Attia A Attia Alzahrani

**Affiliations:** 1 Department of Microbiology, Al-Baha University, Al-Baha, SAU; 2 Department of Parasitology, Al-Azhar University, Cairo, EGY; 3 Department of Physiology, Al-Baha University, Al-Baha, SAU; 4 Department of Dermatology, Al-Baha University, Al-Baha, SAU; 5 Department of Internal Medicine, Al-Baha University, Al-Baha, SAU; 6 College of Medicine, Al-Baha University, Al-Baha, SAU; 7 Department of Microbiology, Al-Baha Central Lab, Ministry of Health, Al-Baha, SAU

**Keywords:** al-baha province, complications, distension, entamoeba histolytica, gastroenteritis, prevalence, ulcer

## Abstract

Background: *Entamoeba histolytica* is a pathogenic protozoan parasite that can cause amoebiasis, which is one of the leading causes of death from parasitic infections.

Aims: This study aimed to estimate the prevalence of intestinal amebiasis among Saudi patients who attended the two major hospitals, including King Fahad Hospital in Al-Baha and Prince Meshari Hospital in Baljurashi, which is a big governorate in Al-Baha, in addition to the Central Laboratory in the Al-Baha region.

Methods: This is a retrospective study of demographic, clinical, radiological, and laboratory data collected from patients' records covering the period from January 2019 to December 2024.

Results: Out of a total of 6,471 stool samples examined, 534 (8.25%) were positive for *E. histolytica* in the form of cysts or trophozoites. Of these positive cases, 305 (57.1%) occurred in males, while 229 (42.9%) were in females. The highest proportion of infections was detected among children aged 6-12 years, accounting for 173 cases (32.4%). In terms of geographic distribution, the majority of cases were recorded in Al-Baha city (296 cases; 55.4%), followed by Baljurashi (195 cases; 36.5%), with the lowest prevalence observed in being from Ghamed and Hajra governorates. Stool macroscopic examination findings included blood in stool in 76 (14.2%) samples of undigested food in 134 (25.1%) samples. Microscopy findings included cysts in 518 (97%) samples, trophozoites in 130 (24.3%), white blood cell (WBC) count of more than 76 cells/μL in (31.5%) 168 samples, and red blood cell (RBC) count of more than 26 cells/μL in 59 (11%) samples. Presence of blood in stool was strongly associated (p < 0.001) with trophozoite. Semi-formed, watery, and mucoid stools were significantly associated with trophozoites. Count of RBCs of >26 cells/μL was significantly (p < 0.001) associated with trophozoite infections. Colonoscopy findings were significantly (p < 0.001) associated with trophozoite cases. Radiological findings were significantly (p < 0.001) higher in trophozoite-positive cases, complications were significantly (p < 0.001) higher in trophozoite cases, and mean corpuscular volume (MCV) was significantly (p = 0.028) lower in cystic cases (75.68 ± 9.7) than in trophozoites (79.44 ± 7.51).

Conclusions: *E. histolytica *remains an important protozoan infection in Saudi Arabia, particularly among the age group of 6-12 years old. The predominance of the cystic form underscores the potential for transmission, and co-infections suggest gaps in sanitation infrastructure. Efforts are needed to evaluate the *E. histolytica *health burden in Al-Baha provinceto enhance the treatment of infected people at primary care centers and to reinforce hygiene.

## Introduction

*Entamoeba histolytica *(*E. histolytica*) is a protozoan causing colonic amebiasis as well as extra-intestinal manifestations. Although 90.0% *E. histolytica* infections are asymptomatic, 50 million people become symptomatic, with about 100,000 deaths annually, and so it is considered the third cause of death from parasitic infections [1]. Amoebic liver abscess formation is a common complication that develops in the right lobe of the liver and is the most common extraintestinal complication. It can be seen from months to years after colonic exposure to infection [2]. Respiratory tract and cardiac brain infection complications are rare complications [1].

*E. histolytica* is prevalent worldwide and higher in countries with low socioeconomic status and poor hygienic habits. Countries with a high rate of infections include Africa, India, Mexico, and South America [3]. In Bangladesh, a study among three-year-old preschool children showed that *E. histolytica* accounted for 2.2% of dysentery cases [1]. As high as 42.0% *E. histolytica *seroprevalence has been reported in rural areas of Mexico [4]. Factors that increase mortality and complicated infections include young age, corticosteroid treatment, malignancy, malnutrition, pregnancy, and alcoholism [1]. Fulminant amoebic colitis is associated with 40% mortality [5].

In Saudi Arabia, high rates of infection with intestinal parasitic diseases among food handlers (14%), expatriates (55.7%), the Abha community (13.2%), Riyadh school children (14.2%), and patients attending hospitals (31.3%) have been reported [6]. Elsewhere in Saudi Arabia, *E. histolytica* was identified in 4.7% of hospital attendees in Makkah [7] and in 20% of infants and children aged 1 month to 16 years who presented with gastroenteritis in Jeddah [8]. A prevalence of *E. histolytica* infection was reported in 9.3% of intestinal parasitic infections [9] and in 53.3% of individuals with intestinal parasites in the Riyadh region [10]. Additionally, the parasite was detected in 2.7% of food handlers in the southern part of Saudi Arabia and in 16.15% of both symptomatic and asymptomatic individuals in Hail [11]. Consequently, *E. histolytica* is an important contributor to intestinal parasitic infection despite the prevalence variation, likely due to geographical and population group variations, which may reflect variation in hygiene standards. This disparity prompts studies to be conducted in more geographical locations to contribute to understanding the big picture of the burden of infection in the country at large. Additionally, to the best of our knowledge, the studies on *E. histolytica* in Al-Baha remain scarce. Therefore, this study investigates the prevalence of the associated clinical, radiological, and hematological manifestations. Consequently, *E. histolytica* remains an important contributor to intestinal parasitic infections, despite variations in its prevalence, likely due to geographical differences and population groups. The variation can also reflect variation in hygiene standards, as the low prevalence among food handlers suggests. This disparity prompts studies to be conducted in additional geographical locations to contribute to understanding the broader picture of the infection burden across the country. Therefore, this study investigates the prevalence of intestinal amebiasis and sociodemographic factors and clinical indicators among patients who attended healthcare facilities.

## Materials and methods

Study populations

The population of these governorates was estimated to be over 314,532 people, representing about 91% of the total population of Al-Baha Province in Saudi Arabia [12]. We used Raosoft sample size calculator (Raosoft, Inc., Seattle, WA, USA) to calculate the sample size, with a 95% confidence interval (CI) and a 0.05 margin of error. As there were no previous studies in Al-Baha on the prevalence of *E. histolytica* infection, we assumed the expected population proportion to be 0.5. The resulting sample size needed was 384, and the final sample size was 534. This study utilized a cross-sectional design and retrospectively assessed the prevalence of intestinal amoebiasis among Saudi people living in the Al-Baha region, which is located in the southwestern Hejaz region of Saudi Arabia. The data for the study were collected from patients' files from January 2019 to December 2024 in King Fahad Hospital, Prince Meshari Hospital, and the Central Laboratory in Al-Baha after obtaining ethical approval from the Research Ethics Committee at the Faculty of Medicine in Al-Baha University (REC/MIC/BU-FM/2022/52).

Data collection

Data for the study were collected retrospectively from patients' files through a data source survey. The survey encompassed a wide range of information, including sociodemographic variables such as age, sex, and residency, as well as various clinical indicators such as stool consistency, color, presence of blood and mucous, white blood cell (WBC) count, red blood cell (RBC) count, presence of cysts and trophozoites, food ingestion patterns, presence of other infections, and radiological investigations. In addition, stool specimens were processed and examined using standard parasitological techniques, including direct wet mount preparation, saline concentration, and formol-ether or zinc sulfate flotation concentration methods in some cases, to diagnose *E. histolytica* based on the clinical manifestations.

Statistical analysis

IBM SPSS Statistics for Windows, Version 21 (Released 2013; IBM Corp., Armonk, New York, United States) was used in this study for data processing and analysis. The data were presented as mean ± standard deviation (SD) or standard error of the mean. To assess differences between groups, one-way analysis of variance (ANOVA) was employed, and we used the chi-squared test for statistical analysis, with p <0.05 set as the level of significance. We also used multivariable logistic regres­sions analysis to assess the association between the prev­alence of *E. histolytica* and potential risk factors.

## Results

**Figure 1:**
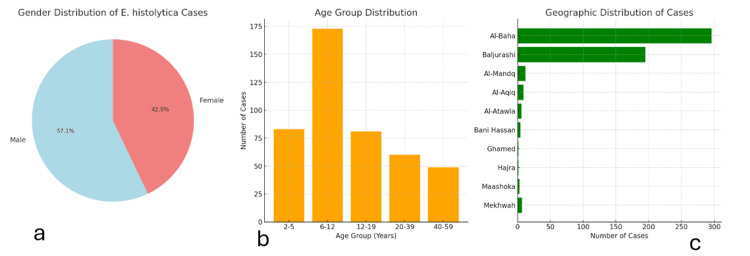
Visual representation of the key demographic data for patients infected with Entamoeba histolytica. (a) Gender distribution: Males account for a higher percentage of *E. histolytica* infections (57.1%) compared to females (42.9%). (b) Age group distribution: Children aged 6-12 are the most affected group. (c) Geographic distribution: Most infections occur in Al-Baha city and Baljurashi.

Of the 6,471 stool samples investigated, 534 (4.7%) were positive for *E. histolytica*. These cases were from patients with a mean age of 26.53 ± 25.77 years and included 305 (57.1%) males and 229 (42.9%) females, with the highest rates observed in children aged 6-12 years and residents of Al-Baha city, followed by those living in Baljurashi (Table 1 and Figures 1a-1c).

**Table 1:** Demographic characteristics of patients infected with Entamoeba histolytica.

Characteristics	Variable	Number	%
Gender	Male	305	57.1
	Female	229	42.9
Age	Group 1 (2-5)	83	15.5
	Group 2 (6-12)	173	32.4
	Group 3 (12-19)	81	15.2
	Group 4 (20-39)	60	11.2
	Group 5 (40-59)	49	9.2
	Group 6 (60 and more)	88	16.5
Residence	Al-Aqiq	9	1.7
	Al-Atawla	6	1.1
	Al-Baha	296	55.4
	Al-Mandq	12	2.2
	Baljurashi	195	36.5
	Bani Hassan	4	0.7
	Ghamed	1	0.2
	Hajra	1	0.2
	Maashoka	3	0.6
	Mekhwah	7	1.3

The stool samples were semi-formed in 240 cases (44.9%), watery in 222 (41.6%), yellow in 137 (35.0%), brown in 181 (33.9%), and green in 136 (25.5%), with 76 (14.2%) containing blood and 134 (25.1%) showing undigested food. A total of 168 samples (31.5%) contained >76 WBCs/μL, 193 (36.1%) had 5-10 RBCs/μL, 404 (75.7%) contained vegetative forms, and 518 (97.0%) were positive for cysts. Co-infections included nonspecific bacteria in 30 samples (5.6%), *Escherichia coli* in 11 (2.1%), *Ascaris lumbricoides* in one (0.2%), *Enterobius vermicularis* in one (0.2%), and yeast in five (0.9%) (Table 2).

**Table 2:** Macroscopic and microscopic findings of examination of stool samples from patients infected with Entamoeba histolytica.

Findings			Number	%
Macroscopic findings	Consistency of stool	Formed	49	9.2
		Semi-formed	240	44.9
		Watery	222	41.6
		Mucoid	20	3.7
		Bloody	3	0.6
	Stool colour	Black	6	1.1
		Brown	181	33.9
		Green	136	25.5
		Red	22	4.1
		Tarry	2	0.4
		Yellow	187	35
	Blood	Null	458	85.8
		Present	76	14.2
	Indigested food	Null	400	74.9
		Present	134	25.1
	Cysts	Null	16	3
		Present	518	97
Microscopic findings	Coinfections	Ascaris lumbricoides	1	0.2
		Bacterial infection (non-specific)	30	5.6
		Escherichia coli	11	2.1
		Enterobius vermicularis	1	0.2
		Null	486	91.1
		Yeast	5	0.9
	WBC	1-15 cell/μL	123	23
		16-75 cell/μL	134	25.1
		More than 76 cell/μL	168	31.5
		Null	109	20.4
	RBC	0-5 cell/μL	50	9.4
		11-25 cell/μL	148	27.7
		5-10 cell/μL	193	36.1
		More than 26 cell/μL	59	11
		Null	84	15.7
	Vegetative form (trophozoite)	Null	404	75.7
		Present	130	24.3

**Figure 2:**
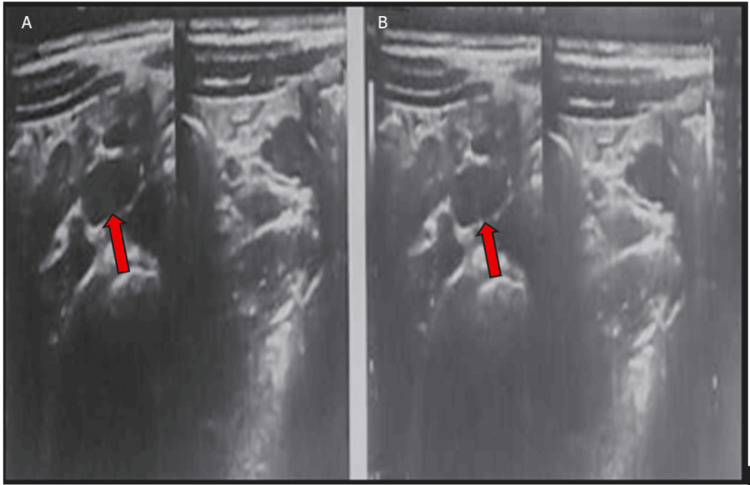
(A, B) Abdominal ultrasound images with the red arrows indicate dilated bowel loops containing echogenic foci of gaseous distension.

**Figure 3:**
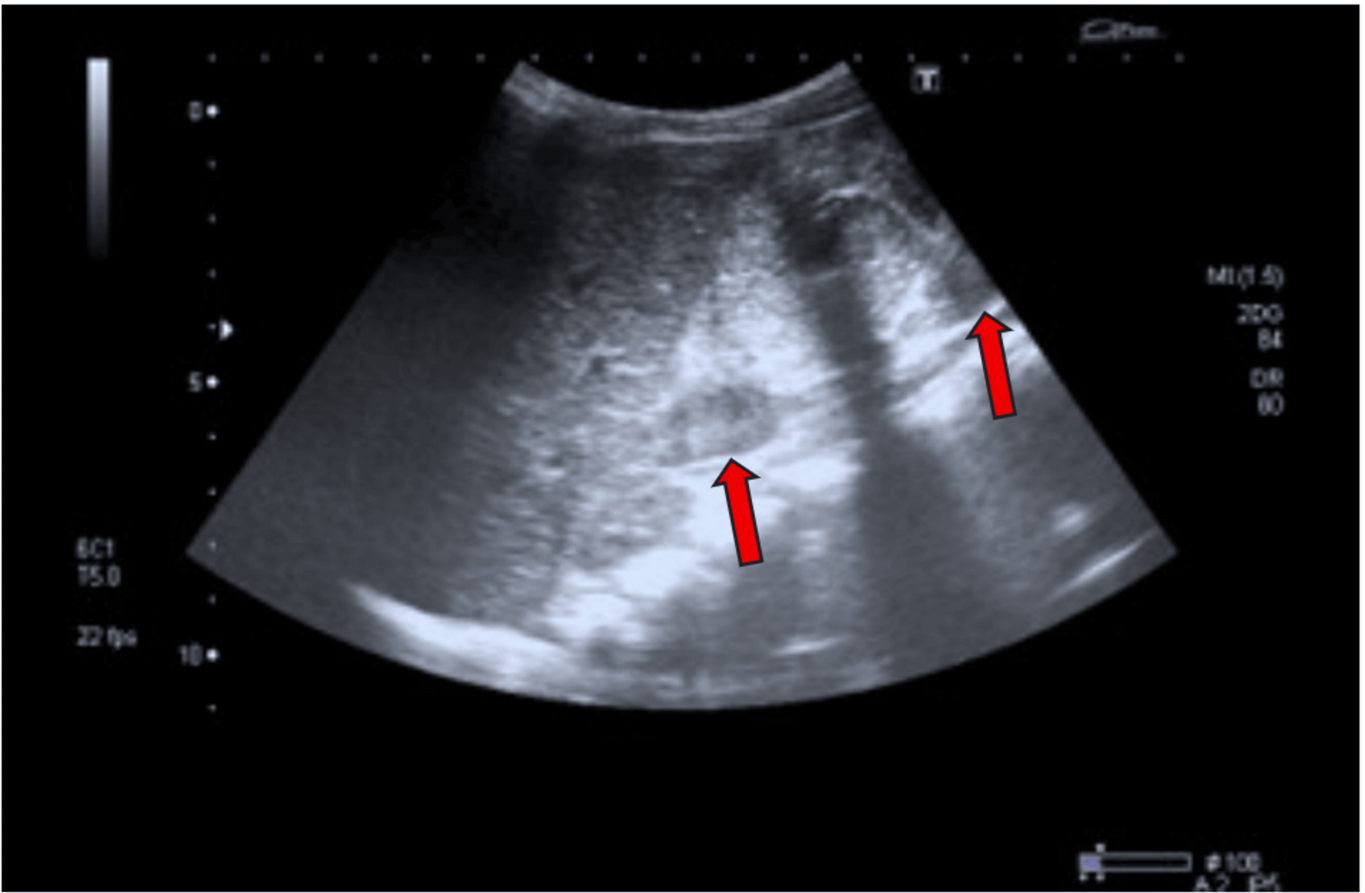
Ultrasound image showing bilateral mesenteric lymph nodes (LNs), measuring 13 × 6 mm on the right side and 17.4 × 4.7 mm on the left side (red arrows).

Ultrasound revealed a colonic mass in one patient (0.2%), which was confirmed by CT scan; enlarged mesenteric lymph nodes were observed in two patients (0.4%); and dilated bowel with distension and edematous thickened walls was noted on ultrasound in 13 patients (1.9%) (Table 3 and Figures 2-3). Complications included bouts of severe diarrhea in 25 patients (3.8%), mucoid diarrhea in 20 (3.7%), diarrhea alternating with constipation in six (1.2%), tarry stool in two (0.4%), and severe anemia in one patient (0.2%) (Table 3).

**Table 3:** Radiological and associated clinical findings among patients infected with Entamoeba histolytica (n = 534). US: ultrasound; CTW: colonic thick wall; ML: mesenteric lymph

Category	Finding	Number	%
Radiology	Colonic mass US (CT abdomen revealed a 3.4 cm mass in diameter)	1	0.2
	CTW/distension	4	0.8
	Enlarged ML US	2	0.4
	Dilated bowel US	2	0.4
	Distension, edematous, and thick-walled US	13	1.9
	Null	194	36.3
Complications	Bloody diarrhea	3	0.6
	Diarrhea alternates with constipation	6	1.2
	Colonic mass	1	0.2
	Boats of severe diarrhea	25	3.8
	Mucoid diarrhoea	20	3.7
	Tarry stool	2	0.4
	Severe anemia	1	0.2
	Null	477	89.3

**Figure 4:**
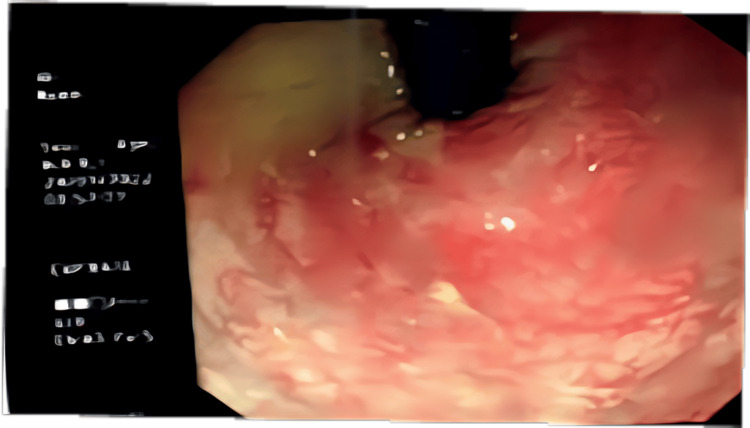
Colonoscopic image demonstrating mucosal hyperemia characterized by diffuse erythema and increased vascular pattern indicate inflammatory colitis.

**Figure 5:**
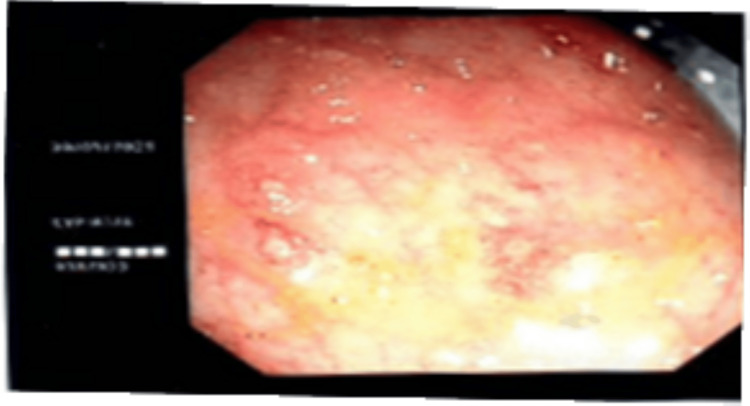
Colonoscopic image showing colonic ulcerations with areas of mucosal erythema and exudative changes.

Colonoscopic findings in 175 cases (32.7%) revealed colonic hyperemia in one case (0.2%), colonic ulcers and mucosal erosions in 21 cases (3.9%), and large intestinal stricture in one case (0.2%) (Figures 4-5).

Of the 305 males, 233 (76.4%) had cysts and 72 (23.6%) had trophozoites, while among 229 females, 171 (74.7%) had cysts and 58 (25.3%) had trophozoites (p = 0.90). The presence of trophozoites was significantly associated with watery stool (p < 0.01), higher red blood cell count (p < 0.001), complications (p < 0.001), visible blood (p < 0.001), and undigested food (p < 0.001). In contrast, cysts were more common in semi-formed stools (p < 0.001) and were associated with moderate white blood cell counts (p < 0.01), while other findings showed no significant associations (p > 0.05) (Table 4).

**Table 4:** Findings of stool examination of stool samples that were positive for cyst and trophozoite. WBC: white blood cell; RBC: red blood cell

Examination	Category	Cyst, n (%)	Trophozoite, n (%)	P-value
Consistency of stool	Formed	35 (8.7)	14 (10.8)	>0.05
	Semi-formed	204 (50.5)	36 (27.7)	<0.001
	Watery	153 (37.9)	69 (53.1)	<0.01
	Mucoid	11 (2.7)	9 (6.9)	<0.05
	Bloody	1 (0.2)	2 (1.5)	>0.05
WBC	Null	33 (8.2)	12(9.2)	>0.05
	1-15 cells/μL	93(23)	30 (23.1)	>0.05
	16-75 cells/μL	113 (28)	21 (16.2)	<0.01
	>76 cells/μL	43 (10.6)	12 (9.2)	>0.05
RBC	Null	63 (15.6)	21 (16.2)	>0.05
	5-10 cells/μL	165 (40.8)	28 (21.5)	<0.001
	11-25 cells/μL	106 (26.2)	42 (32.3)	>0.05
	>26 cells/μL	32 (7.9)	27 (20.8)	<0.001
Radiology	Finding	13 (7.7)	9 (10.9)	>0.05
	No finding	118 (92.3)	73 (89.1)	
Complications	Complications	27 (6.7)	30 (23.1)	<0.001
	No complications	377 (93.3)	100 (76.9)	
Colonoscopy	Finding	19 (16.7)	14 (23)	0.3112
	No Finding	95 (83.3)	47 (77)	
Blood	Present	31 (7.7)	45 (34.6)	<0.001
	Null	373 (92.3)	85 (65.4)	
Mucus	Present	326 (80.7)	108 (83.1)	>0.05
	Null	78 (19.3)	22 (16.9)	
Indigested food	Present	63 (15.6)	71 (54.6)	<0.001
	Null	341 (84.4)	59 (45.4)	

Hematological parameters, including hemoglobin, RBC indices, and total and differential WBC counts, did not differ significantly (p > 0.05) between patients with cysts and those with trophozoites (Table 5).

**Table 5:** Hematological findings among patients infected with Entamoeba histolytica. WBC: white blood cells; HB: hemoglobin; PCV: packed cell volume; MCV: mean corpuscular volume; MCH: mean corpuscular hemoglobin; MCHC: mean corpuscular hemoglobin concentration; PLT: platelet count; RDW: red cell distribution width; lymph: lymphocytes; mono: monocytes; Esino: eosinophils; Baso: basophils; SD: standard deviation

Parameter	Cystic forms, mean ± SD	Trophozoite, mean ± SD	P-value	Significance
WBC	10.1 ± 4.92	9.3 ± 5.9	0.513	>0.05
HB	12.07 ± 1.93	12.66 ± 2.15	0.195	>0.05
PCV	36.55 ± 5.03	37.77 ± 5.69	0.306	>0.05
MCV	75.68 ± 9.7	79.44 ± 7.51	0.028	< 0.05
MCH	25.53 ± 2.82	26.96 ± 4.28	0.100	>0.05
MCHC	32.96 ± 1.58	33.43 ± 1.42	0.134	>0.05
PLT	364.37 ± 115.76	351.79 ± 140.97	0.667	>0.05
RDW	14.14 ± 1.62	14.39 ± 2.97	0.669	>0.05
Neutrophils	53.41 ± 22.33	53.27 ± 22.19	0.976	>0.05
Lymph	36.26 ± 21.28	35.3 ± 20.97	0.832	>0.05
Mono	9.39 ± 5.19	9.44 ± 5.25	0.965	>0.05
Esino	1.43 ± 1.52	1.74 ± 2.37	0.507	>0.05
Baso	0.34 ± 0.25	0.67 ± 3.26	0.605	>0.05

## Discussion

The current study showed that the prevalence of *E. histolytica* in the Al-Baha region of Saudi Arabia is 8.3%, which falls within the global range of infection, especially in developing countries where sanitation and hygiene standards may vary. Similar studies have been conducted in other regions of the country. For example, Jeddah, Saudi Arabia, has reported comparable prevalence rates ranging from 4% to 12% [13] and 1.6% in Riyadh [14]. Regarding gender distribution, the infection was more prevalent among males (305 cases; 57.1%) compared to females (229 cases; 42.9%). This gender disparity is often attributed to behavioral and occupational exposure, where males may be more likely to engage in outdoor activities or consume food from less hygienic sources [15]. The highest rate of infection was found in children aged 6-12 years (173 cases; 32.4%), followed by the 2-5 age group (83 cases; 15.5%). This aligns with global epidemiological trends showing increased susceptibility among children, possibly due to underdeveloped immune systems, higher exposure to contaminated environments, e.g., schools, public playgrounds, and the lack of hygiene in children [16]. Most *E. histolytica* cases originated from Al-Baha city (296 cases; 55.4%) and Baljurashi (195 cases; 36.5%). These areas likely have higher population densities, which may increase transmission opportunities. In contrast, rural areas such as Ghamed and Hajra reported very low numbers (one case each; 0.2%), potentially due to smaller populations or better sanitation practices in those households.

Regarding morphological forms, a large majority (518 cases; 97%) of infections were in cystic form, whereas trophozoites were identified in only 130 samples (24.3%). Cystic forms are more resilient and transmissible, while trophozoites indicate active infection. The lower trophozoite detection rate could be due to sample collection timing, as trophozoites degrade rapidly outside the host [17]. The study found co-infections, primarily with non-specific bacterial pathogens (5.6%) and *E. coli* (2.1%), alongside a few helminthic parasites like *A. lumbricoides *and *E. vermicularis*. These findings suggest possible fecal-oral transmission due to poor hygiene or inadequate water treatment [18]. Semi-formed stools characteristics were the most common (44.9%), followed by watery stools (41.6%). This indicates a classical manifestation of *E. histolytica* infections [19]. Blood and mucus presence, while less frequent, highlight the potential for invasive disease. Additionally, the presence of undigested food in 25.1% of cases may reflect gastrointestinal malabsorption, potentially linked to *E. histolytica* pathogenicity. Complications included mucous diarrhea in 3.7% and severe watery diarrhea in 2.6% of cases, with blood detected in 5.1%. Cellular analysis showed that a significant proportion of samples contained >26 RBCs/µL (11%) and >76 WBCs/µL (31.5%), supporting the diagnosis of inflammatory intestinal conditions commonly associated with invasive amebiasis [1]. Significant associations were found between trophozoite presence and watery stools (p < 0.01) or mucoid stools (p < 0.05). Semi-formed stools were more commonly associated with cyst carriers, while blood in stool was significantly associated with trophozoite presence (p < 0.001), suggesting mucosal invasion. Undigested food was also significantly more frequent in trophozoite carriers (54.6%) compared to cyst carriers (15.6%) (p < 0.001), likely due to impaired digestion from intestinal inflammation [20]. These findings support that trophozoites, rather than cysts, are responsible for active and symptomatic disease. White and red blood cells in stool were significantly associated with trophozoite presence: WBCs (16-75/μL, p < 0.01) and RBCs (>26/μL, p < 0.001), indicating that mucosal invasion by trophozoites leads to colitis with leukocyte and erythrocyte exudation, consistent with an inflammatory response [21].

Radiological findings in our study showed that, of the 213 patients (39.8%) who underwent ultrasound (US), a colonic mass was identified in only one patient (0.2%) and confirmed by CT (3.4 cm in diameter). Enlarged mesenteric lymph nodes (MLNs) were found in two cases (0.4%), dilated bowel/distension was detected in six cases (1.2%), and edematous, thickened bowel walls were observed in 13 cases (1.9%). These findings, though present in a small percentage, are clinically significant. Thickened bowel walls and lymphadenopathy are signs of active inflammation or possible complications like ameboma formation (a mass-like lesion due to chronic *E. histolytica* infection) [17]. In the present study, colonoscopy was performed in 175 patients (32.7%). Findings included colonic ulceration/mucosal erosion in 21 cases (3.9%), colonic hyperemia in 11 cases (2.1%), and large intestinal stricture in one case (0.2%). These results are consistent with invasive amebiasis, where *E. histolytica* trophozoites invade the colonic mucosa, causing ulcerations, bleeding, and potentially strictures [22]. Complications were significantly more frequent in trophozoite carriers (23.1%) compared to non-trophozoite carriers (6.7%; p < 0.001). Severe diarrhea occurred in 3.8% of cases, mucoid diarrhea in 3.7%, and anemia or bloody/tarry stools were observed in a few severe cases. These complications are consistent with manifestations of invasive amebiasis, particularly in immunocompromised or pediatric patients [23]. Hematological variables due to the effect of *E. histolytica* infections in our study showed no significant differences between cyst and trophozoite carriers, but mean corpuscular volume (MCV) was significantly lower in cyst infections (p < 0.05), suggesting possible microcytic anemia in chronic asymptomatic infections. Hemoglobin and WBCs were slightly lower in trophozoite carriers, but differences were not statistically significant; this implies that systemic inflammation or anemia is not a dominant feature in uncomplicated intestinal amebiasis, unless complicated by invasive disease [24].

The parasite that causes amoebic dysentery infections is usually transmitted by eating contaminated food. The greatest way to prevent the transmission of this disease is to wash hands thoroughly with soap and running water after using the bathroom and before eating. Good hygiene is the key to preventing amoeba or dysentery, and there are a number of practices and procedures that can achieve prevention, including washing vegetables and fruits with vinegar or peeling well before eating them. Drinking bottled water is important, and canned soft drinks. If you need to drink tap water, boil it first and treat it with a little iodine [25].

Despite these findings, this study has certain limitations. The cross-sectional study limits causal inference, and the use of microscopy alone may have caused misclassification since it cannot differentiate other types of amoeba, but associated clinical manifestations suggest* E. histolytica *infections. The strengths of this study are through large sample size, which provides strong statistical power and enhances the reliability of the prevalence estimates. Also, data were collected from multiple sources of major hospitals and the central laboratory, which increases the representativeness of the sample, and the study included demographic, clinical, radiological, colonoscopy, and laboratory data, giving a multidimensional view of the disease and its presentation. Future research using molecular diagnostics and larger population-based samples with more colonoscopic and radiological data is recommended.

## Conclusions

*E. histolytica* is an important protozoan infection among preschool children. The predominance of the cystic form underscores its potential for transmission, and co-infections indicate gaps in sanitation infrastructure. Efforts are needed to evaluate the health burden of *E. histolytica* in Al-Baha province to improve treatment of infected individuals at primary care centers and to reinforce hygiene. Consequently, screening of school children and adults, promotion of health education, and raising awareness among the Saudi population and educational staff about *E. histolytica* infections and their prevention remain necessary.
